# Effects of ultrasonic treatment on ovomucin: Structure, functional properties and bioactivity

**DOI:** 10.1016/j.ultsonch.2022.106153

**Published:** 2022-09-07

**Authors:** Qi Xu, Xuanchen Li, Yunzheng Lv, Yaping Liu, Chunfang Yin

**Affiliations:** a Institute of Advanced Cross-Field Science, College of Life Science, Qingdao University, Qingdao, Shandong Province 266800, China; b College of Food Science and Engineering, Northwest A&F University, No. 22 Xinong Road, Yangling, Shaanxi 712100, China

**Keywords:** Ovomucin, Ultrasound, Solubility, Function properties, Bioactivity

## Abstract

The effects of ultrasonic treatment on the structure, functional properties and bioactivity of Ovomucin (OVM) were investigated in this study. Ultrasonic treatment could significantly enhance OVM solubility without destroying protein molecules. The secondary structure changes, including β-sheet reduction and random coil increase, indicate more disorder in OVM structure. After ultrasonic treatment, the OVM molecule was unfolded partially, resulting in the exposure of hydrophobic regions. The changes in OVM molecules led to an increase in intrinsic fluorescence and surface hydrophobicity. By detecting the particle size of protein solution, it was confirmed that ultrasonic treatment disassembled the OVM aggregations causing a smaller particle size. Field emission scanning electron microscopy (FE-SEM) images showed that ultrasonic cavitation significantly reduced the tendency of OVM to form stacked lamellar structure. Those changes in structure resulted in the improvement of foaming, emulsification and antioxidant capacity of OVM. Meanwhile, the detection results of ELISA showed that ultrasonic treatment did not change the biological activity of OVM. These results suggested that the relatively gentle ultrasound treatment could be utilized as a potential approach to modify OVM for property improvement.

## 1 Introduction

In the past decade, extensive attention has been paid to bioactive proteins and peptides extracted from food. As an important source of dietary protein, egg white plays a unique role in food industry. OVM belonging to the mucin family, is an essential linear sulphate glycoprotein in egg white that maintains the gel properties of fresh egg white. It mainly exists in thick protein, accounting for 2 %–4% of total protein [1]. Previous studies have confirmed that OVM and its hydrolysates have a variety of biological activities, including antiviral [2], anti-tumor [3], antibacterial [4] and anti-inflammation [5]. Nevertheless, according to its complex physicochemical properties, The potential of OVM in the biomaterials and food industries has not been fully exploited.

In general, enzymatic hydrolysis or chemical modification to proteins is a common way to improve the properties of proteins. However, the classical chemical modification process is usually complex. On the other hand, the chemical reactions in the process may result in the residue of toxic substances, leading to potential safety concerns [6]. Although enzymatic hydrolysis can partially destroy the structure of proteins to produce some biologically active fragments, the generation of bitter peptides is a major disadvantage that can not be ignored [7]. Physical methods are generally considered safe and environment-friendly to improve protein function [8]. Thus, physical modification is advantageous over other techniques in treating proteins and peptides.

As a new physical processing technology, ultrasonic has attracted extensive attention for its unique mechanical characteristics [9]. High-intensity ultrasound mainly affects the whole system through cavitation and microstreaming currents. As sound waves propagate through the water medium, the tiny bubbles led by acoustic waves will implode in the liquid system. The strong hydrodynamic shear forces, pressures, and temperatures generated from this processing can alter protein properties [10], [11]. So far, evidence has confirmed that ultrasonic treatment can change the structure and properties of proteins, such as reducing particle size, changing secondary structure, and changing solubility [12]. However, excessive ultrasonic treatment will cause protein destruction research has reported that excessive ultrasound processing broke the structures of jackfruit seed protein [13]. Therefore, moderate ultrasonic treatment is considered a potential protein modification approach.

In this study, the OVM was treated with an ultrasound probe for different times. Ultrasonic treatment markedly increased the solubility of OVM in water. The structural characterization of the protein showed that ultrasound changed the secondary and tertiary structures of OVM. The extension of the protein structure led to the exposure of the hydrophobic groups, increasing intrinsic fluorescence and surface hydrophobicity. Meanwhile, ultrasound effectively reduced the aggregations of protein in the solution. In addition, the changes in physicochemical properties improved the emulsifying property, foaming property and antioxidant activity of OVM. ELISA assay confirmed that gentle ultrasound treatment did not damage the bioactivity of OVM. The results will expand the application of OVM in biological materials and functional food.

## 2 Materials and methods

### 2.1 Materials

The isolation and purification of OVM were obtained by ClearFirst-2000 protein purification system (Shanpu, Shanghai, China) [14]. Egg and maize oil were purchased from a local market. NaCl was obtained from Sinopharm (Sinopharm Chemical Reagents Co. Ltd, Shanghai, China). Gel chromatography column packing material Sepharose® CL-6B was purchased from Sigma reagents company (Sigma-Aldrich, Missouri, US). 2,2′-Azino-bis(3-ethylbenzothiazoline-6-sulfonic acid) diammonium salt (ABTS), Potassium persulfate, 2,2-Diphenyl-1-picrylhydrazyl (DPPH), and 8-Anilino-1-naphthalene sulfonic acid (ANS) were purchased from Macklin (Macklin Biochemical, Shanghai, China). All chemicals and solvents used in this work were analytical reagents.

### 2.2 Ultrasonication of OVM

OVM was stirred overnight in borate-hydrochloric acid buffer (0.02 M, pH = 8.6) at 4 °C to fully hydrate. A Model IID ultrasonic probe (Scientz, Ningbo, China) was used to sonicate OVM at ice-bath (power: 100 W, pulse duration: 1 s) for different times (from 0 to 40 min). Samples were transferred to centrifugal tubes, and all of them were centrifuged by a high-speed refrigerated centrifuge for 30 min at 15000 rpm. The supernatant of all groups was collected separately. The concentration was determined by BCA method and diluted slightly for further study.

### 2.3 Solubility

10 mg OVM was put into 10 mL double distilled water per group. Total five groups were prepared and stirred overnight at 4 °C for complete hydration. An ultrasonic probe was used to sonicate all groups. Furthermore, every group was centrifuged to collect supernatant. Then the concentration of the supernatant was detected by BCA method using a NanoDrop One (Thermo Fisher, Massachusetts, US). every group was repeated three times and solubility of each group was calculated by the following formula:

$$\text{Soulbility}=\frac{W_{2}}{W_{1}}\times 100\text{\textbackslash{}\%}\;$$

Here, W_1_ – total weight of OVM, W_2_ – weight of OVM in supernatant.

### 2.4 Native polyacrylamide gel electrophoresis (Native-PAGE)

A Native-PAGE was applied to analyze the molecular structures of the untreated and sonicated OVM. The precast BeyoGel™Plus PAGE (Beyotime Biotechnology Co. Ltd, Shanghai, China) included 4 % stacking gels and 15 % separating gels. 80 μL of sample was mixed with 20 μL loading buffer (4 × ). Gel board was mounted into a Biorad Mini-PROTEAN Tetra Electrophoresis System (Biorad, California, US), and 20 μL of each sample mixture was loaded into gel lanes. The running buffer was prepared by Tris-Gly Native-PAGE buffer powder. A color mixed protein marker of 11–180 kDa (Solarbio, Beijing, China) was utilized to estimate the molecular mass of each sample. The whole electrophoresis process kept the operating voltage at 150 mV.

### 2.5 Sodium dodecyl sulfate–polyacrylamide gel electrophoresis (SDS-PAGE)

SDS-PAGE was applied to analyze the molecular structures of the untreated and sonicated OVM. The polyacrylamide gel was prepared using an SDS-PAGE gel kit (5 % stacking gels and 8 % separating gels). Each sample was mixed with 80 μL borax-hydrochloric acid buffer solution (0.02 M, pH = 8.6) and 20 μL loading buffer (4 × ). All samples were put in a boiling water bath for 10 min then cooled to room temperature. Gel board was mounted into a Biorad Mini-PROTEAN Tetra Electrophoresis System, and 20 μL of each sample mixture was loaded into gel lanes. Color mixed protein standard of 11–180 kDa was applied for estimations of the molecular mass of each sample. The operating voltage during stacking stage process was 80 mV and 120 mV in separating stage.

### 2.6 Intrinsic fluorescence emission spectrum

All samples were diluted with borax-hydrochloric acid buffer (0.02 M, pH = 8.6) to 0.5 mg/mL. The fluorescence spectrum was measured by an FS5 fluorescence spectrophotometer (Edinburgh Instruments, Edinburgh, UK) at room temperature. All samples were tested at Ex = 295 nm. The excitation slit and emission slit width were both 2 nm. The recording range of fluorescence spectrum was from 300 to 450 nm.

### 2.7 Surface hydrophobicity

The surface hydrophobicity was determined according to the previous method with slight modification [15]. Briefly, All sample solution was diluted to 0.3 mg/mL at 25 °C. After that, 20 μL ANS (8 mM) was added to 4 mL of each sample solution. The mixtures were led to react 15 min away from light. Then fluorescence emission spectra of the mixture were recorded with a fluorescence spectrophotometer at Ex = 390 nm.

### 2.8 Circular dichroism analysis (CD)

The samples were returned to room temperature and diluted to 0.3 mg/mL with borax-hydrochloric acid buffer (0.02 M, pH = 8.6). In addition, the CD spectrum of all samples was obtained via a J-810 spectropolarimeter (Jasco, Tokyo, Japan) at 25 °C. The range of spectroscopy records was from 190 to 240 nm. Meanwhile, the scanning speed was 100 nm/min with 1 nm bandwidth during the process [16].

### 2.9 Particle size

The particle size of all samples was measured by a ZS90 Malvern Zetasizer (Malvern Instruments, Worcestershire, UK), referring to the previous method [17]. The sample solution was returned to room temperature in advance and diluted to 0.5 mg/mL with borax-hydrochloric acid buffer (0.02 M, pH = 8.6). The measurement results were based on dynamic light scattering (DLS).

### 2.10 Field emission scanning electron microscopy (FE – SEM)

The microtopography of samples was observed by GeminiSEM 500 scanning electron microscopy (ZEISS, Oberkochen, Germany). The samples were stuck to the surface of the conductive tape and covered with gold. The microtopography was observed in a 2.0 kV acceleration voltage.

### 2.11 Emulsifying property

Emulsifying activity index (EAI) and emulsion stability index (ESI) of samples were examined with the turbidimetric method [18]. Firstly, the mixture containing 10 mL maize oil and 40 mL sample solution (1 mg/mL) were homogenized by an FJ200-SH high-speed homogenizer (Huxi, Shanghai, China) at a speed of 12000 rpm for 1 min. Then, 100 μL of emulsion were pipetted out successively from beaker bottom at 0 min and 30 min. The emulsion were added into 2 mL of 0.1 % (w/v) sodium dodecyl sulfate (SDS) solution. The absorbance was determined at 500 nm (0.1 % SDS as blank) by a UV-2600 spectrophotometer (Shimadzu, Tokyo, Japan). EAI and ESI were gotten by the following formulas:

$$\mathrm{EAI}\;\left(m^{2}/g\right)=\frac{2\times 2.303\times A_{0}\times DF}{c\times \emptyset \times 10000\times \theta }$$

$$\mathrm{ESI}\;\left(\min\right)\;=\frac{A_{0}}{A_{0}-A_{30}}\;\times \;30$$

where A_0_ and A_30_ are the absorbance of mixture at 0 and 30 min. DF represents the dilution factor; **∅**represents the path length of the cuvette (cm); C is the protein concentration (g/m^3^) and θ is the volume fraction of oil.

### 2.12 Foaming property

Foam expansion (FE) and foam stability (FS) of samples were tested by previous mothed [17]. Briefly, 30 mL of sample solution was transferred into 50 mL measuring cylinders and homogenized by a high-speed homogenizer at 12000 rpm for 1 min. The total volume of the system was recorded at the point of 0 and 30 min.

$$\mathrm{FE}\;\left(\%\right)=\frac{V_{0}}{V_{o}}\times 100$$

$$\begin{array}{c} FS\;\left(\%\right)=\frac{{\text{V}}_{30}}{{\text{V}}_{o}}\times 100 \end{array}$$

where V_O_ is the original volume; V_0_ is the volume after homogeneity and V_30_ is the volume after 30 min.

### 2.13 ABTS radical scavenging assay

The ABTS radical scavenging activities of samples (1 mg/mL) were carried out by the method described in the previous study [19]. The ABTS was diluted to the absorbance at 734 nm was 0.7 ± 0.02 as the working solution. Then 200 μL ABTS working solution and sample 20 μL were added to the 96 well plate. The absorbance of mixture was monitored at 734 nm with a SuPerMax 3100 microplate reader (Shanpu, Shanghai, China) after 6 min. The control was prepared by replacing the sample with water. Free radical scavenging activity (%) of sample was calculated using the following formula:

$$\mathrm{ABTS}\;scavenging\;ability\;\left(\%\right)\;=\left(1-\frac{A_{b}-A_{a}}{A_{b}}\right)\times 100$$

Where A_a_ was the absorbance of mixture of samples and ABTS solution, A_b_ was the absorbance of mixed solution of deionized water and ABTS solution.

### 2.14 DPPH radical scavenging assay

The DPPH radical scavenging activities of samples (1 mg/mL) were determined using a previously described method [20]. Specifically, in 96 well plate, 20 μL of each sample was added to 200 μL of DPPH solution (0.2 mM in ethanol). The mixture was incubated for 30 min away from light at 25 °C. A microplate reader measured the absorbance of all groups at 517 nm. The control was prepared by replacing the sample with deionized water. The scavenging activity (%) of the sample was calculated using the following formula:

$$\mathrm{DPPH}\;scavenging\;activities\;\left(\%\right)\;=\left(1-\frac{A_{a}-A_{b}}{A_{b}}\right)\times 100$$

Where A_a_ was the absorbance of mixture of samples and DPPH solution, A_b_ was the absorbance of mixed solution of deionized water and DPPH solution.

### 2.15 Enzyme linked immunosorbent assay (ELISA)

All samples were diluted to 50 μg/mL, and the diluted solution was added to 96-well plate with 100 μL/well. The 96-well plate was stored at 4 °C overnight. Then the protein solution was removed. The wells were washed with PBST (1 × ) 3 times. Then, 5 % skim milk was added to a 96-well plate with 300 μL/well and incubated at 37 °C for 1 h for blocking. After the plates were washed, OVM antibody (CWBIO, beijing, China) diluted 1:80000 was added at 100 μL/well, and the plates were incubated at 37 °C for 1.5 h. After the plates were washed, HRP-labeled goat anti-mouse IgG (CWBIO, beijing, China) diluted 1:8000 was added at 100 μL/well, and the plates were incubated at 37 °C for 80 min, washed and stained.

### 2.16 Statistical analysis

Experiments were carried out in triplicate and all the obtained data was presented as mean ± standard deviation. For pair comparison, one-way analysis of variance (ANOVA) was used, and significance of difference was established at p < 0.05. Statistical analysis was performed using SPSS 12.0 software (SPSS Inc., Chicago, IL, USA).

## 3 Results and discussion

### 3.1 Solubility of OVM

**Fig. 1:**
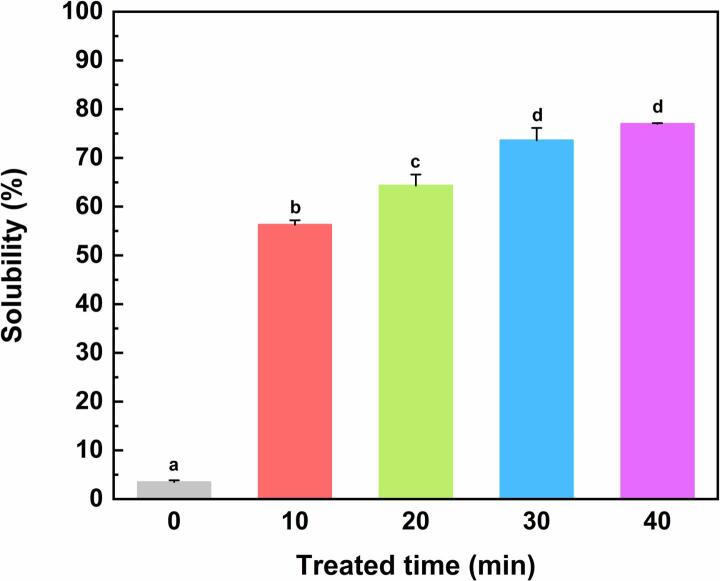
Solubility in water of untreated and sonicated OVM. Different superscript letters in the figure denote significant differences (p < 0.05).

**Fig. 2:**
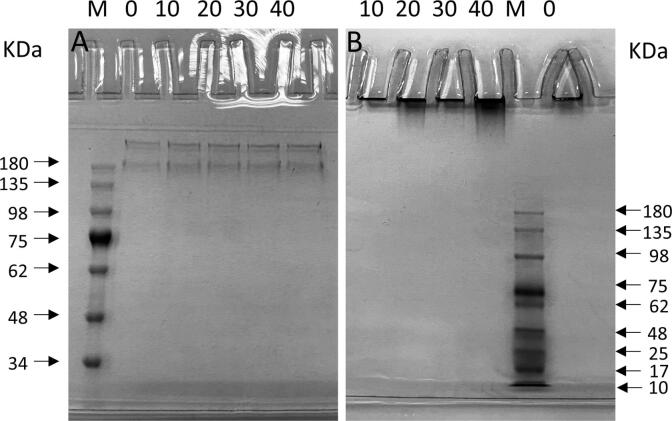
Effects of ultrasound treatment on the molecular weight of OVM. SDS-PAGE (A) and Native-PAGE (B).

The OVM concentration in supernatant was determined by the BCA method, and the solubility of samples was calculated by formula. OVM was generally dissolved in alkaline buffer with high ionic strength for the poor solubility in water. The effect of ultrasonic treatment on the solubility of OVM in water is shown in Fig. 1. The solubility was remarkably promoted from 3.50 ± 0.37 % to 56.29 ± 0.91 % after 10 min ultrasound treatment. The solubility continued to increase with the extension of treatment time. A previous study on egg white proteins (EWP) found that high-intensity ultrasound could increase the solubility of EWP by degradation of OVM [21]. However, in this work, no evidence showed that OVM trended to degrade significantly by ultrasound (Fig. 2). Thus, solubility improvement of OVM was most likely influenced by two factors. First, ultrasound could break up protein aggregations by cavitation [22]. The destruction of the aggregations facilitated the contact of the hydrophilic groups in protein molecules with water [22]. Secondly, the shift in molecular structure might enhance the interaction between OVM and water, thus increasing solubility.

### 3.2 Electrophoretic analysis

Fig. 2A shows SDS-PAGE of OVM under different ultrasound times. All samples showed a consistent pattern of α subunit band near 180 KDa and β subunit band above 180 KDa. It suggested that the molecular weights of the α and β subunits of OVM did not change significantly [23]. No significant differences or new bands appeared in every treated group compared to the native OVM. The results confirmed that ultrasonication did not make remarkable cleavage in the molecular structure of OVM. The same phenomenon was also reflected in Native-PAGE results (Fig. 2B). The intact OVM was challenging to pass through the stacking gels for its large molecular weight, so there would be no bands in the separation gel. All treated groups accumulated proteins at the top of the lane, but no bands were found in separating gel. Those indicated that OVM was not breakage under these sonication conditions, and the overall structure of OVM has not disintegrated. Similarly, previous studies verified that no molecular weight alterations appeared in squid mantle proteins and whey protein isolate after ultrasound treatment [24].

### 3.3 Surface hydrophobicity

**Fig. 3:**
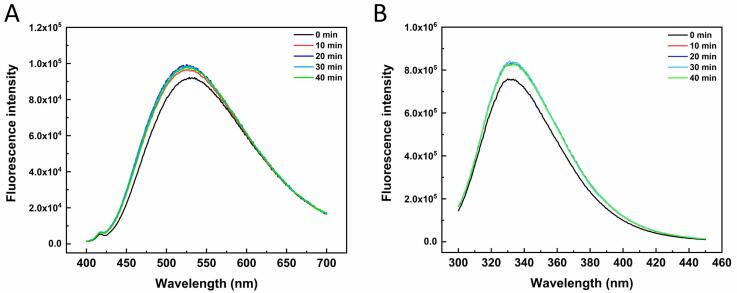
Effects of ultrasonic treatment on surface hydrophobicity (A) and intrinsic fluorescence (B) of OVM molecular.

ANS is used to detect the surface hydrophobicity of proteins by binding with high affinity to the hydrophobic surface of the protein. Fig. 3A shows the fluorescence intensity changes of sample solution after mixing with ANS. The fluorescence intensity increased after ultrasonic treatment, and the fluorescence intensity of treatment groups increased first and then decreased with time. For native proteins, most hydrophobic groups were wrapped within the protein molecule. Some of the hydrophobic groups in OVM molecules could be exposed during the ultrasonic processing, which generated the rise of surface hydrophobicity [25]. Furthermore, natural proteins tend to form aggregations in solution, and the formation usually relies on the binding of hydrophobic regions between protein molecules [26]. The large aggregation was broken down into smaller ones by the mechanical forces, thus freeing more hydrophobic areas and increasing surface hydrophobicity. The transformation of secondary structure often alters protein surface hydrophobicity. Ultrasound treatment increased the α-helix content and decreased the β-sheet content of OVM. The increase in α-helix and decrease in β-sheet content might indicate a loosening of the protein molecule and exposure of more hydrophobic groups [27]. Meanwhile, the variation of OVM secondary structure with time was consistent with surface hydrophobicity (Table 1). Therefore, it could be considered that the OVM molecule exposed the hydrophobic groups inside and increased its surface hydrophobicity.

**Table 1:** Secondary structure changes of OVM after different treated times.

Samples	α-helix (%)	β-sheet (%)	β-turn (%)	Random coil (%)
0 min	4.16 ± 0.49^c^	71.53 ± 0.12^a^	1.56 ± 0.24^d^	22.90 ± 0.24^c^
10 min	4.63 ± 0.12^c^	69.16 ± 0.61^b^	2.33 ± 0.16^bc^	23.86 ± 0.55^bc^
20 min	6.46 ± 0.16^a^	63.66 ± 0.38^d^	3.06 ± 0.20^a^	26.80 ± 0.71^a^
30 min	6.06 ± 0.44^ab^	64.66 ± 1.14^c^	2.76 ± 0.12^ab^	26.50 ± 0.57^a^
40 min	5.53 ± 0.20^b^	67.46 ± 0.85^d^	2.16 ± 0.28^c^	24.83 ± 0.77^b^

Different letters indicate significant differences (p < 0.05).

### 3.4 Intrinsic fluorescence emission spectrum

Intrinsic fluorescence spectroscopy usually refers to the fluorescence emission of tryptophan or tyrosine residues in proteins. It is regarded as a common way to evaluate the conformational changes of proteins, especially the tertiary structure changes. All samples' fluorescence spectra were obtained using tryptophan as an internal fluorescent probe. As shown in Fig. 3B, similar to previous studies [28], ultrasonic treatment could change the intensity of protein intrinsic fluorescence peak but not the maximum emission wavelength of protein solution. Compared to native OVM, the intrinsic fluorescence intensity of all treated groups significantly increased. The fluorescence intensity increase reflected the protein structure changes, indicating the OVM molecule unfolding. The structure changes and the rupture of hydrophobic interactions by ultrasound resulted in more hydrophobic groups being exposed to the OVM surface [27]. Interestingly, all treated groups showed no significant differences in fluorescence intensity, unlike the reduction in intrinsic fluorescence caused by high power or long treatment time [29]. This phenomenon could be explained by the ultrasound condition (100 W, within 40 min) did not appear to reduce fluorescence intensity though guiding protein reaggregation.

### 3.5 Particle size

**Fig. 4:**
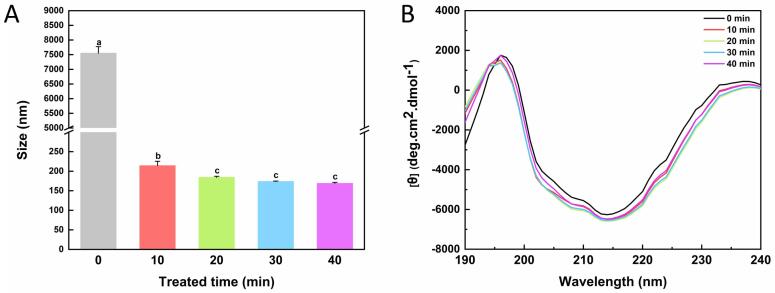
Effects of ultrasonic treatment on average particle size (A) and secondary structure (B) of OVM molecule. Different superscript letters in the figure denote significant differences (p < 0.05).

The particle size in protein solution is related to the functional properties of proteins. Therefore, the effect of ultrasonic treatment on the size of OVM particles was evaluated using dynamic light scattering (DLS). As shown in Fig. 4A, untreated OVM exhibited a particle size over 7000 nm but decreased rapidly to 214.26 ± 11.26 nm after 10 min ultrasonic treatment. In addition, with the increase in ultrasonic process time, the particle size of OVM further decreased to 184.35 ± 2.86 nm and finally stabilized at about 170 nm. Compared to the native OVM, the particle size was significantly reduced after ultrasonic treatment. Previous studies reported that 200 W ultrasound treatment within 30 min could reduce the particle size of chicken plasma protein in solution by disrupting protein aggregations [30].

At first, OVM was fully hydrated in solution to form the large network aggregations that appeared as a thin gel in macroscopic. The complex network exhibited a large particle size. Under mechanical forces, the van der Waals force was broken, causing large OVM aggregations to be shattered into smaller pieces [31]. The protein molecule's structure will fully adjust through the physically induced stretching vibration. Therefore, the particle size of OVM decreased after ultrasonic treatment. Like an ultrasound study on soy protein isolate [32], the mean diameter of the OVM decreased significantly during the first 20 min and remained constant between 20 and 40 min. These results indicated that the longer ultrasonic treatment did not cause further changes in OVM particle size under this condition. In addition, the small particle morphology may significantly impact the functional activity of OVM.

### 3.6 Circular dichroism analysis

Circular dichroism is a common method to measure the secondary structure of proteins. The secondary structure of OVM after ultrasound pretreatment is shown in Fig. 4B and Table 1. The results showed that the α-helix and β-turn of the OVM were decreased, while β-sheet and random coil were increased by ultrasound compared with natural protein. This trend reached its maximum at 20 min, after which the extension of ultrasound time led to a rebound. The ultrasonic study of Whey protein emulsion gels produced similar changes in α-helix and β-sheet [33]. Those changes in OVM might cause the protein molecule to unfold, which may result in the exposure of the hydrophobic part of the protein interior [34]. The result was consistent with the increase in surface hydrophobicity and intrinsic fluorescence. The secondary structure changes were attributed to the cavitation force of ultrasound, which disrupted the interaction between protein molecules and affected the internal structure of protein molecules [30]. Notably, similar to previous studies onovalbumin [35], low-intensity ultrasound could alter the tertiary structure of OVM but have little effect on the secondary structure. It may be explained that ultrasound primarily disrupts protein–protein interactions without violently altering the secondary structure of the protein. Thus, similar to previous trends, the ultrasound-treated groups showed greater agreement on the spectrum.

### 3.7 FE-SEm

**Fig. 5:**
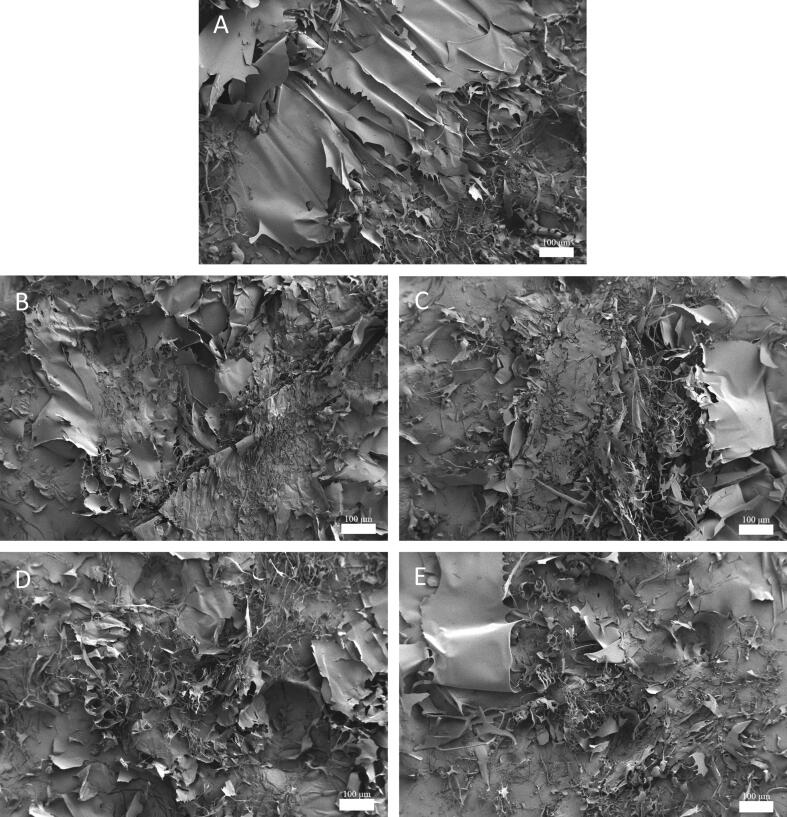
Morphology of OVM determined by scanning electron microscopy. Untreated (A); 10 min (B); 20 min (C); 30 min (D); 40 min (E).

FE-SEM carried out the morphology of each group to evaluate the impact of ultrasound treatment on OVM. In Fig. 5, The images revealed that freeze-dried native OVM tended to form multilayer sheet structures. In contrast, the trend was significantly reduced after 10 min ultrasound treatment, and the collapse of lamellar structures was observed. Furthermore, with the extension of ultrasonic time, OVM presented fragmentary morphology with different sizes. Analogously, whey protein isolate (WPI) was broken down into smaller pieces after ultrasound treatment [36]. Like WPI, the ultrasonic probe reduced the particle size of OVM by breaking up the protein aggregations with the shock wave. This effect broke the entanglement between OVM molecules and caused the network structure to collapse. The degradation of stacked lamellar structure was also observed in morphology. Interestingly, the OVM treated by ultrasound was more readily redissolved than natural OVM. So, the destruction of OVM aggregations may positively affect its dissolution.

### 3.8 Foaming properties assay

**Fig. 6:**
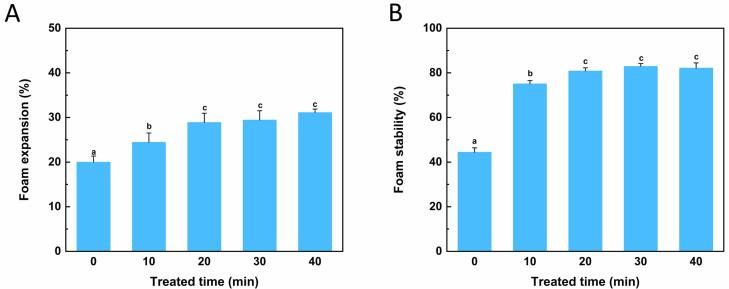
Effects of ultrasonic treatment on foam properties of OVM. Foam expansion (A) and foam stability (B). Different superscript letters in the figure denote significant differences (p < 0.05).

The foam is usually defined as the dispersion of bubbles in a liquid, where a layer of liquid separates the trapped gas. The foaming property is one of the functional properties for proteins, related to the film film-forming capacity of protein solution at the air–liquid interface. Usually, foam expansion (FE) and foam stability (FS) are two indexes to evaluate the foaming properties. Fig. 6 shows the FA and FS of OVM. As expected, the FA value of OVM increased from 20.00 ± 1.36 to 24.4 ± 2.07 after 10 min ultrasound treatment and further increased to about 31.11 ± 0.78 after 40 min treatment. Similar to perious studies on *Moringa Oleifera* seed protein, the ultrasound could enhance the foaming ability of the protein [37]. Ultrasound improved the solubility of OVM, increasing soluble protein, which allows more protein adsorbed at the gas–liquid interface and enhances its foam ability. In addition, the partial unfolding of OVM exposed more hydrophobic groups to the surrounding environment, improving the surface activity [38]. The FS value reflected the same trend as FA. Partial denaturation of OVM molecules during ultrasound might be a reason for the enhancement of FS value. The stability of a bubble was often related to the thickness of the layer separating the air. Unfolding of OVM molecules results in forming a thick, sticky layer around the bubble, which improves foaming stability [39]. Further, a previous study on egg yolk showed that higher ultrasound power could decrease foam stability [37]. Nevertheless, in this work, the treatment under ultrasound did not cause similar effects in OVM, which may be related to the power and mode of ultrasound.

### 3.9 Emulsifying properties assay

**Fig. 7:**
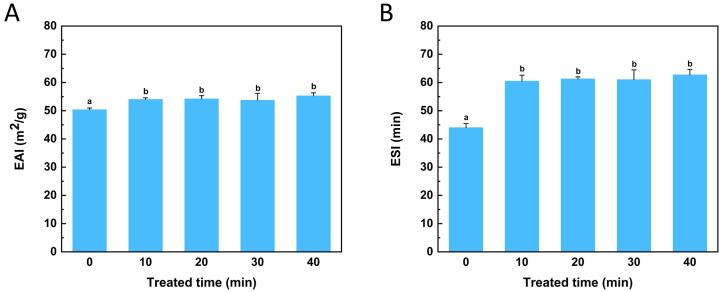
Effects of ultrasonic treatment on emulsion properties of OVM. Emulsion activity index (A) and emulsion stability index (B). Different superscript letters in the figure denote significant differences (p < 0.05).

Protein emulsion is a semi-solid mixture that plays a vital role in the food industry. Protein particles are adsorbed on the surface of the oil droplets to form a protective layer, keeping the droplets away from coalescence, flocculation and gravity separation [40]. As shown in Fig. 7, EAI of the treated group slightly increased compared with native OVM, while ESI significantly increased. Previous studies showed that surface hydrophobicity, particle size, and solubility could affect the emulsifying properties by affecting protein adsorption, concentration and diffusion rate at the water–oil interface [41]. The emulsifying capacity of native OVM was slightly lower than that of the treated groups. This result might be due to the destruction of OVM aggregations by homogenization mechanical forces. Compared with the treated groups, the ESI of natural OVM appeared significant decrease. It could be interpreted as the surface hydrophobicity of treated groups increased, making it more stable to adsorb on the water–oil interface. This effect could reduce the interfacial tension between two phases and improve the emulsion stability.

Meanwhile, flexibility is a factor that affects the emulsifying properties of proteins. After ultrasonic treatment, the OVM molecular was partially unfolded, leading to increased interactions between proteins and lipids, as well as protein rearrangements at the oil–water interface [42]. Flexible protein molecule resulted in higher surface hydrophobicity and solubility, enhancing its ability to stabilize the oil–water interface. The enhancement of protein solubility can increase the protein distribution at the oil–water interface, thus forming a thicker interface layer. At the same time, the increase of surface hydrophobicity can promote the interaction between protein molecules in the interface layer, thus increasing the density of the interface layer and improving its emulsification [42]. Ultrasound could reduce the aggregation of proteins and improve surface activity, thus making the droplets of protein emulsions smaller and more stable [43]. The disintegration of OVM aggregations may produce smaller lipid droplets to reduce the release rate of emulsion, thus improving the stability of the emulsion.

### 3.10 Antioxidant activity assay

**Fig. 8:**
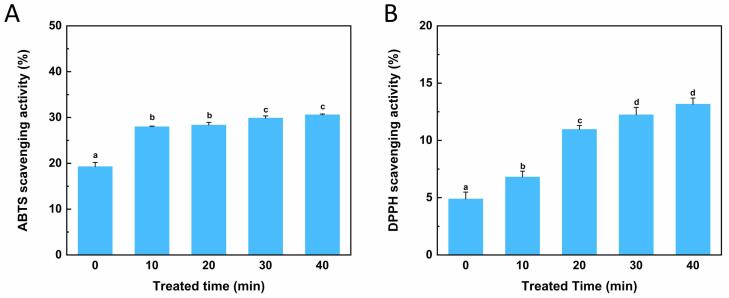
Changes in antioxidant activity of OVM after different ultrasonic treatment time. ABTS scavenging rate (A) and DPPH scavenging rate (B). Different superscript letters in the figure denote significant differences (p < 0.05).

Earlier studies showed that ultrasound could enhance the antioxidant capacity of casein and whey proteins. Therefore, ABTS and DPPH were used to evaluate the antioxidant activity of OVM after ultrasonic treatment. Fig. 8 reflected the scavenging activity of all samples to ABTS and DPPH free radicals. As expected, similar to previous studies [44], natural OVM showed poor antioxidant activity. However, compared with OVM without ultrasonic processing, the ultrasonic treatment effectively increased the antioxidant activity of the treated group. Although, the scavenging ability of DPPH was lower than that of ABTS, which may be due to the ethanol. Studies showed that some amino acid residues, such as aromatic and sulfur-containing amino acids, can exert antioxidant functions by supplying protons to free radicals [45]. After ultrasonic treatment, protein unfolding led to a partial expansion of the molecular structure. Antioxidant amino acid residues were exposed during this process and more readily contacted with free radicals, improving the antioxidant activity of OVM. In addition, the peptides with small molecular weight and hydrophobic end tended to have stronger ABTS free radical scavenging ability [46]. Therefore, it could be inferred that the size reduction and the surface hydrophobicity increase of OVM after ultrasonic treatment may also be the reason for improving antioxidant activity.

### 3.11 ELISA assay

**Fig. 9:**
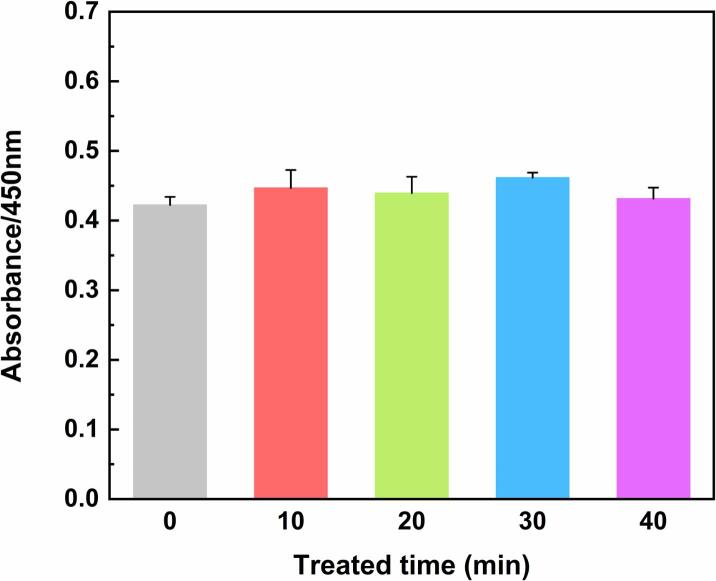
Antibody binding ability of OVM after ultrasonic treatment with different times (p > 0.05).

The binding ability between antibody and receptor is one of the important indexes for evaluating bioactivity. The effect of ultrasound treatment on the biological activity of OVM was detected by ELISA. As shown in Fig. 9, compared with native OVM, the absorption value of ultrasonic treatment group at 450 nm was slightly improved. However, prolonged ultrasound time did not significantly change the absorption value of OVM. Evidence verified that ultrasound could enhance the binding of IgG and IgE to OVA [47]. Ultrasound could expose more sites by loosening the ovalbumin structure and breaking down protein aggregations. Analogously, changes in the structure and particle size of OVM under ultrasound treatment exposed hidden sites, which led to a slight increase in the ability of binding antibodies. However, the improvement effect was not significant between treated groups, which was also consistent with changes in the structure and particle size of OVM. These results indicated that the bioactivity of OVM was not changed under the ultrasound condition of this work.

## 4 Conclusion

In this research, OVM was treated with ultrasound for different times, and the effects of ultrasound on its structure, function and other characteristics were investigated. Due to linear structure and complex carbohydrate chains, OVM was more likely to lead to complex network aggregations than other spherical proteins. Ultrasonic treatment could break the aggregations of OVM in solution. During the ultrasonic treatment, the structure of protein molecules tends to partially unfold, increasing the interaction between the protein and water. In terms of results, ultrasound affected the secondary and tertiary structure of the OVM and changed the surface hydrophobicity. Those changes led to the rise of solubility and improved oxidation resistance, foaming, and emulsifying properties. In addition, the ELISA assay showed that ultrasound treatment did not significantly change the bioactivity of OVM. Interestingly, unlike proteins treated by a higher power ultrasound, prolonged treatment time with low power (especially after 10 min) did not significantly affect the OVM. Therefore, this work would extend the theoretical basis for OVM changes under ultrasound and its potential application in the food industry.

## CRediT authorship contribution statement

**Qi Xu:** Funding acquisition, Conceptualization, Supervision, Writing – review & editing. **Xuanchen Li:** Investigation, Writing – original draft. **Yunzheng Lv:** Investigation, Validation. **Yaping Liu:** Resources, Formal analysis. **Chunfang Yin:** Software, Validation.

## Declaration of Competing Interest

The authors declare that they have no known competing financial interests or personal relationships that could have appeared to influence the work reported in this paper.

## Data Availability

Data will be made available on request.
